# Machine Learning and RSM for Lattice Structure Optimization

**DOI:** 10.3390/polym18050627

**Published:** 2026-03-03

**Authors:** Giampiero Donnici, Marco Freddi, Leonardo Frizziero

**Affiliations:** Department of Industrial Engineering, Alma Mater Studiorum—University of Bologna, v.le Risorgimento 2, 40136 Bologna, Italy; giampiero.donnici@unibo.it

**Keywords:** response surface method, artificial neural networks, lattice structures, stiffness optimization, resin additive manufacturing

## Abstract

This study concerns the analysis of lattice structures printed with EPAX resin for the manufacturing of a motorcycling throttle cam with Response Surface Methodology (RSM) and Artificial Neural Networks (ANNs). The design of the pattern core in the lattice structure is defined parametrically to identify optimal design points (best stiffness to weight ratio in particular). Some geometric parameters used as input in RSM and in the NN analysis include the origin of the lattice structure and its spatial orientation, cell dimensions, and thicknesses. The dataset obtained with this approach is used for an RSM analysis of variance (ANOVA) to highlight the most important inputs. NN analysis is performed on the same RSM dataset to confirm the results. Both methodologies identify in-domain points of optimal design due to the typical non-linear behavior of these structures. The literature and industrial experience already provide numerous references to studies characterizing lattice structures. However, related practical applications are often incomplete and only achieve functional rather than optimal models. The approach described also aims to overcome this limitation. The software used for the design is nTop 5.0.4.

## 1. Introduction

Lattice structures [1] are generally characterized by repetitive patterns of one or more geometrical cells distributed inside the volume of a mechanical part [2,3,4]. Their use is aimed at structural lightening (keeping the external shape of the geometries unchanged) with a general improvement in mechanical properties [5,6] or at improving heat transfer properties [7]. There are many types of cells already known to the scientific community and industrial design. The “classic” porous structures are composed of patterns of beams with constant or variable thickness distributed within a cell. The most used are cubic (as body centered or face centered), diamond, fluorite, octet, Weaire–Phelan, IsoTruss, Kelvin cell and others [8,9,10,11]. They are generally easy to print with FDM or SLM 3D printers and good for reducing the relative density. Usually they deform/offer resistance along preferential direction.

In the literature there are several studies related to RSM and ANN methodologies defining the architecture of lattice structures and their optimization [12,13]. For example, the energy absorption capacity in structural crashes is a typical object of study [14,15,16]. Areas of interest range from the industrial sector [17,18,19] to the biomedical one [20], considering plastic materials [21], cement [22] or metallic materials [23] printed with different techniques, demonstrating the multi-applicability of these structures. The same print parameters have been subject to RSM analysis [24]. The field of composites is often involved in RSM studies [25]. Their application can predict the behavior of new materials considering the variability of base materials, geometries, loads and constraints [26]. Several multi-objective studies in thermodynamics have also been performed with RSM to optimize the thermal capabilities of heat exchangers [27,28] or other devices useful for CO_2_ absorption [29].

Concerning machine learning (ML), anisotropy studies performed with Convolutional Neural Networks (CNNs) integrated with homogenization allow the mapping of the relationships between geometries and structural performance [30,31], conducting to their optimization [32,33]. Structural improvements were obtained with studies of variability of trabecular thickness in cells thanks to hybrid neural networks [34]. New models, such as 3DGAN, have been implemented to parametrically approach the design of a lattice structure improving its extension capacity and absorption energy [35]. Challapalli et al. worked on a reverse machine learning approach to increase the compressive stress capabilities of sandwiches with excellent results [36]. Optimizing printing parameters is often addressed with ML studies to overcome the limitations of regression models [37]. Here, the application sector ranges from that of mold making [38] to that of industrial devices [39] and optimization software [40]. These works collectively underscore the transformative impact of ML in redefining the boundaries of lattice structure design and optimization, and they seem to work better than models based on polynomial regressions or DOE-RSM [32,41]. However, the main limitation associated with the integration of ML is related to the large amount of data required to train neural networks. Prototyping times linked to 3D printing of complex geometries, coupled with test realization times, can often prove to be excessive [42,43]. Frequently, the use of established methodologies such as Design of Experiments (DOE), constructed on the minimum number of trials necessary for the study of the system, remains probably favorable.

Although the literature provides numerous references to types, data, and properties of lattice structures, their practical application still proves challenging. The choice of cell type to employ frequently depends on the shape of the component and the applied loads. When the optimal cell type is known with certainty, understanding the optimal values for cell dimensions remains a complex task. For these reasons, the following study proposes an approach aimed at obtaining a reliable methodology to assess the variation in structural stiffness as the main representative parameters of the model change. These parameters include the size, the beam thickness, the type of cell used and the origin of the pattern and its angular orientation. The reliability of the derived RSM model is verified by comparing its results with those obtained from every single FEM (or experimental) calculation applying random variable cell dimensions, orientations and thicknesses of the constituent beam elements. Following this application, an analysis of the local error obtained from the application of the RSM methodology concerning the variation of cell size is conducted. This variable is continuous and therefore suitable for the RSM methodology. Then, a neural network was trained with the same database for similar regression studies. The methodology is different, and if it offers similar results to the RSM it can be considered as first confirmation. In Section 2 the description of the parameters used for the case-specific analysis is explained in detail. Section 3 describes a practical application of the two methodologies, RSM and ANN, and their comparison. Some theoretical notes are offered for the two methodologies; however, readers are advised to deepen the theory in the literature. The component used as case study is a throttle cam, as written in the abstract. Finally, the methods and setup for the additive manufacturing of the part are rapidly described (nothing more than traditional steps for 3D printing).

## 2. Materials and Methods

### 2.1. Definition of Core Parameters for Dataset Generation

**Figure 1:**
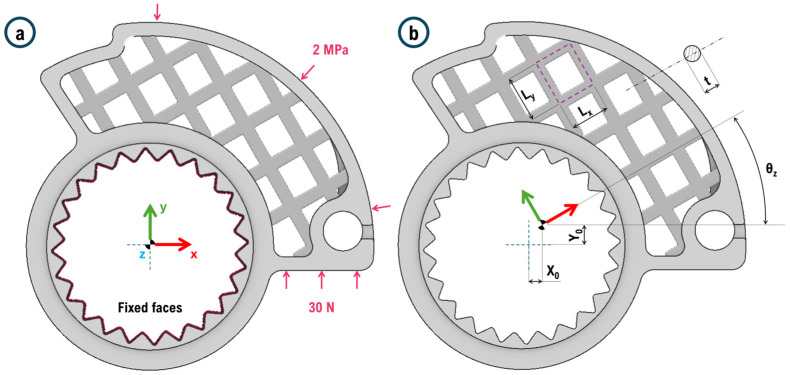
(**a**) Forces and constraints acting on the component; (**b**) parameterization of the core for analysis of variance.

To immediately clarify the parameterization of the core and motivate the definition of some limits in the model, it is advisable to refer to Figure 1. Loads and constraints applied to the throttle cam and the variables used to parameterize the design of the lattice core are schematized. These parameters are used to generate multiple combinations of inputs for the dataset used for RSM-ANOVA and neural network training. The loads applied are based on experience data. The 30 N on the lower face are transferred from the limit switch on the handlebar to the component in the condition of maximum throttle opening. The 2 MPa load on the top face is due to the contact between the wire connected to the engine throttle and the throttle cam in the drive-by-wire system.

Figure 1 shows simple cubic cells even though the study is focused on the use of face-centered cells, as described later. This is to simplify the comprehension of parameters to the readers; it is just a scheme. According to the introductory paragraph, it is quite challenging to choose which lattice cell to use when component lightweighting is required. Designers are familiar with the geometry of the component (typically, the external geometry must remain unchanged in these cases), as well as the constraints and loads. In this study, the initial approach involves identifying the internal volume of the component for lightweighting (the core). This is performed on the only portion of material sufficiently distant from direct contact with a load or constraint. A solid material thickness of 5 mm is maintained along all external edges of the part in line with technical specification. Once the core is identified, a parametric model of geometry is defined to allow for rapid structural modifications and associated FEM simulations. According to the authors, the defined parameters should represent all degrees of freedom of the structure. Considering the pattern in a generic porous core, it is essential to understand which minimal geometric variations can influence the outputs (stiffness, weight, maximum stresses, displacements, deformations, etc.) and their variability range. It is also necessary to define which parameters will not change during the analysis.

Variations are primarily represented by the type of cell. Here, the analysis of just one type of cells (FCC—face-centered cubic) is considered. This type of cell is good to ensure a quite fast 3D printing process, and this limitation favors a higher number of printed and tested specimens. Nevertheless, the application of the proposed method remains general, and the analysis of different cell types can be considered.

As mentioned in the Introduction, cells have specific shapes and relative densities (i.e., the ratio between the density of the cellular lattice sample and the density of the constituent material), and they can be imagined as located into ideal parallelepipeds. Their sides (with lengths L_x_, L_y_, and L_z_) obviously influence stiffness, weight, deformation, and stress. In this case, the dimensional variations in millimeters of the cells are considered within the ranges [4; 12], [4; 12], and [5; 5], respectively, but these values are arbitrary. A minimum dimension of 4 mm and a maximum dimension of 12 mm are imposed on the X-Y plane. Along the Z-axis, the cell dimension is kept constant and equal to 5 mm. This is due to technical constraints related to the assembly of the throttle cam. In fact, the part must be exactly 5 mm wide and cannot extend beyond its maximum envelope. Furthermore, the variation in the number of beams along the Z direction should be avoided, as it involves stiff discontinuities in the solutions. The stiffness function surely exhibits some discontinuities due to the addition of more beam trusses in the lattice, but the RSM methodology cannot predict this kind of behavior, as it is built on polynomial regression (continuity condition is required in the output as previously underlined). For this reason, L_x_ and L_y_ are accepted, while L_z_ is not.

The position of the pattern origin (variables X_0_, Y_0_, and Z_0_) has influence. If the origin shifts, some lattice cells cut at the interface between the core and the boundary material are modified consequently. An incomplete cell certainly alters the local stiffness of the component. Many incomplete cells could largely influence the stiffness of the global structure. Here, the origin is defined in the central groove of the cam (when it is mounted on the motorcycle, this point lies on the handlebar axis, as in Figure 1). In fact, at this point and in the surrounding area, no lattice is generated since this section must remain empty to allow for mounting. The nTop 5.0.4 software used to parameterize the geometry allows for the restriction of lattice generation within predefined volumes (the core). The lattice translations involve only the X and Y directions. The maximum translation value is defined as 12 mm as the pattern repeats beyond these values, if cells have maximum dimensions, offering the same configuration. Along the Z-axis, the origin is not translated to ensure the symmetry of the throttle cam with respect to the X-Y plane in every analyzed configuration. This also reduces the number of specimens to test.

The orientation of the lattice in space is also intuitively significant (angles θ_x_, θ_y_, and θ_z_). Here, only the variation in θ_z_ is considered to ensure symmetry of the component with respect to the X-Y plane. This variation should fall within the range [0°; 90°] in case of purely cubic cells. Beyond these values, the same configurations are obtained (at 91°, the pattern is the same as at 1°). With non-square cells, the variation involves the range [0°; 180°]. Beyond 180°, the pattern remains the same. To set the lattice rotation angle θ_z_, random tangential values are generated within the range [−90°, 90°] ([0°, 180°] range could work too). Again, this limitation ensures symmetry with respect to the X-Y plane of the component. Only a rotation of the pattern around the Z-axis ensures that all configurations meet this condition.

**Figure 2:**
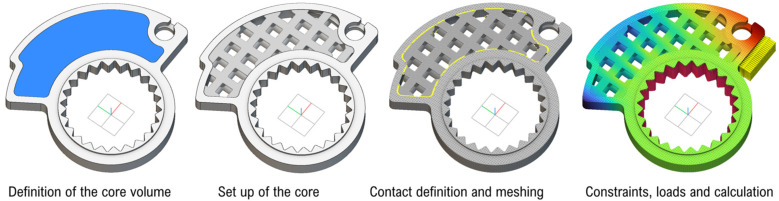
Virtual steps of the throttle cam design (software: nTop 5.0.4).

The average internal thickness (t) may be significant too. It refers to the diameter of the lattice beams into classic cells. This thickness is then varied each time within the range [2; min(L_x_; L_y_)]. A minimum value of 2 mm is imposed to ensure good structural solidity, considering the 0.2 mm thickness of the printing layers (10 layers within the minimum thickness). At the same time, the thickness can theoretically be equal to the smallest side of a cell (in this case it would completely fill the porous pattern in that specific direction, filling the halves of two adjacent cells). Eventually, this condition can be avoided by imposing different limits of variability on the thickness and the cells’ edge length. The main macro-steps of the method are schematized in Figure 2. The next section will be useful for readers to understand them thanks to the description of a real industrial application aimed at the production of a customized component. This is the throttle cam mounted on the handlebars of a motocross motorcycle aimed at adjusting the throttle valve of the engine.

When the parameters are defined, it is possible to generate the database applying simulations and/or physical load tests. The authors believe it is better to start with virtual simulations to find any unsuitable setup. In addition, printing many specimens is time-consuming. The purpose of simulations is not to identify the exact (real) stiffness and displacement values of the component. The goal is in fact to find the most influential variables on the outputs analyzed. This study is about input–output variations, not exact calculations. The database generated from the set of analyzed configurations is then studied with RSM and NN approaches. As written in the introduction, two different approaches on the analysis of the same dataset are useful to verify whether the predictions on new configurations of the lattice core are accurate, validating the two methodologies. This approach is directly described in Section 3 so as not to lengthen the manuscript.

### 2.2. Introduction to ANOVA Methods, RSM, and Input–Output Correlation Analysis

The final database for the ANOVA model can be constructed using several design models. The full factorial approach may be considered, for instance. It simulates every possible combination, taking F factors (inputs), each varying L times (levels), for a total of $F^{L}$. This number rapidly increases, together with costs. A reduction in testing is conceivable with a Central Composite Design (CCD) approach or with the Latin Hypercube Sampling (LHS) one. Reliability decreases consequently.

This procedure is followed once the RSM design is chosen. A high number of simulations are executed to build the dataset. Design variables (inputs) are strategically changed in every simulation, and the performance outputs are measured. After the ANOVA is performed on the database, the final regression equation, table of *p*-values, and Pareto and Normal plots are produced. By highlighting the design elements that have the greatest and least impact, these visual aids help to concentrate the optimization process on the most important variables.

Imagining the output as a linear combination of the inputs, the training process is like system (1) in which, however, the $\beta_{i}$ coefficients are initially unknown:

(1) $$\left\{\begin{array}{c} y_{1}=\beta_{0}+\beta_{1}x_{11}+\beta_{2}x_{12}+\cdots +\beta_{k}x_{1k}+\varepsilon_{1}\\ y_{2}=\beta_{0}+\beta_{1}x_{21}+\beta_{2}x_{22}+\cdots +\beta_{k}x_{2k}+\varepsilon_{2}\\ \vdots \\ y_{n}=\beta_{0}+\beta_{1}x_{n1}+\beta_{2}x_{n2}+\cdots +\beta_{k}x_{nk}+\varepsilon_{n} \end{array}\right.$$

The system can be rewritten in matrix form (2):

(2) $$\begin{array}{l} y=\left[\begin{array}{c} y_{1}\\ y_{2}\\ \vdots \\ y_{n} \end{array}\right]\\ X=\left[\begin{array}{ccccc} 1 & x_{11} & x_{12} & \cdots & x_{1k}\\ 1 & x_{21} & x_{22} & \cdots & x_{2k}\\ \vdots & \vdots & \vdots & \vdots & \vdots \\ 1 & x_{n1} & x_{n2} & \cdots & x_{nk} \end{array}\right] \end{array}$$

Finally, it is possible to calculate the $\beta_{k}$ factors using (3):

(3) $${\left(X^{T}X\right)}^{-1}X^{T}y=\left[\begin{array}{c} \beta_{0}\\ \beta_{1}\\ \dot{\beta_{n}} \end{array}\right]$$

An example of a second-order regression model is given in Equation (4):

(4) $$y=\beta_{0}+\sum_{i=1}^{k}\;\beta_{i}x_{i}+\sum_{i=1}^{k}\;\beta_{ii}x_{i}^{2}+\sum_{i<j}\;{\beta_{ij}x}_{i}x_{j}+\epsilon$$

The weights $\beta_{ij}$ must be identified based on the fit with the point cloud in the database. If the effects of the N factors are only linear, the model can be reduced to a hyperplane. This model easily avoids overfitting, as the curvature of the regression space remains constant and is good for non-linearities. The polynomial enables the identification of optimal or sub-optimal configurations. These configurations are mathematically pinpointed by searching for points with a zero gradient, where the regression model predicts the best performance for the desired outputs (5).

(5) $$\nabla f\left(x_{1},x_{2},\ldots ,x_{n}\right)=\vec{0}$$

where ∇f is the symbol of the gradient, a vector operator that contains all partial derivatives of a function. Then, $f\left(x_{1},x_{2},\ldots ,x_{n}\right)$ is the objective function, and $x_{1},x_{2},\ldots ,x_{n}$ are the input variables, i.e., the suspension design parameters (e.g., kingpin angle, hub length, and so on). More details about DOE, RSM, and ANOVA are in [44].

In summary, this optimization method is not limited to a manual iterative process. It leverages the power of simulations to build predictive models guiding the identification of the most efficient suspension setup, relying on a suite of established software and modeling approaches.

Some indices such as the Pearson correlation coefficient (here defined for a mono-variable analysis for simplicity) is expressed by (6):

(6) $$R^{2}=\frac{\sum_{i=1}^{n}\;\left(x_{i}-\bar{x}\right)\left(y_{i}-\bar{y}\right)}{\sqrt{\sum_{i=1}^{n}\;{\left(x_{i}-\bar{x}\right)}^{2}}\cdot \sqrt{\sum_{i=1}^{n}\;{\left(y_{i}-\bar{y}\right)}^{2}}}=\frac{Cov\left(X,Y\right)}{\sigma_{X}\sigma_{Y}}$$

Here, $x_{i}$ and $y_{i}$ are input and output vectors, $\bar{x}$ and $\bar{y}$ the average values, and n is the number of tests. Since the study is rigorous and impacted by a low computation error rate, high coefficients are expected (even in the 95–98% range).

### 2.3. Introduction to Neural Networks and Main Formulas Used in the Study

The logic by which neural networks learn to predict outputs is similar to the RSM model, but the methodology is different. It involves changing the internal weights of the net during training. In practical terms, neural networks are non-linear structures of statistical data organized as modeling tools. They can be used to simulate complex relationships between inputs and outputs that other analytical functions cannot represent. In this study, supervised learning is applied (i.e., when a training set is available that includes typical examples of inputs with their corresponding outputs). In this way the network can learn to infer the relationship that binds them. Subsequently, the network is trained using a suitable algorithm (typically backpropagation, which is precisely a supervised learning algorithm), which uses this data to modify the weights and other parameters of the network itself in such a way as to minimize the prediction error related to the training set (7):

(7) $$\frac{\partial L}{\partial w_{ij}}=\frac{\partial L}{\partial a_{j}}\cdot \frac{\partial a_{j}}{\partial z_{j}}\cdot \frac{\partial z_{j}}{\partial w_{ij}}$$

Backpropagation applies the chain rule to compute gradients of the loss function L with respect to weights $w_{ij}$ in neural networks. This enables efficient training of deep networks. If the training is successful, the network learns to recognize the unknown relationship that links the input variables to the output variables and is therefore able to make predictions even where the output is not known a priori.

The neural network developed for this study is a Multilayer Perceptron (MLP) with a feedforward architecture, consisting of 6 neurons in the input layer and 1 neuron in the output layer. In fact, there are 6 input parameters for each simulation, and the required output always involves the estimation of one specific value (the ratio between stiffness and weight defined in the next section). Two hidden layers with 10 neurons each are used. The hidden layers utilize Rectified Linear Unit (ReLU) activation functions, selected due to their superior performance in this case compared to sigmoid and tanh, particularly when applied to normalized data in the [0; 1] range. Designed specifically for regression tasks, the network is trained over 450 epochs with a batch size of 45, using the Adaptive Moment Estimation (Adam) optimizer, known for its robustness in handling non-stationary objectives and adaptive learning rates. The learning rate has been set at 0.01 in the internal gradient descent approach (8):

(8) $$\theta_{t+1}=\theta_{t}-\eta \nabla_{\theta }L(\theta )$$

Gradient descent updates parameters (as weights and biases of every node in the NN represented by θ in this equation) by moving opposite to the gradient of the loss function L, scaled by learning rate η. If η=0, obviously weights and biases of the NN will not change during epochs, as weights remain equal to their initial value ($\theta_{t+1}=\theta_{t}=\theta_{0}$). This is the backbone of training in most ML models. Validation is performed every 25 iterations, and the overall training progress is monitored through MATLAB’s built-in visualization tools here. Explicit regularization techniques are not used, as the use of simple architecture reduces the risk of overfitting.

## 3. Results

**Figure 3:**
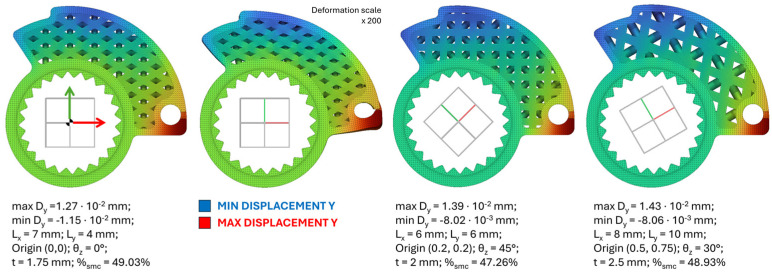
Graph of displacements measured in three case studies of the RSM database.

A series of 250 simulations is conducted in this case study. This number is indicative and can be lower at the expense of the precision and completeness of the database. In general, the more variables analyzed, the higher the number of tests should be. For each setup, the design parameters of the structure and the core are set. These values are randomly generated considering the design limits imposed for each variable (see the previous paragraph). Plausibly accurate values of Young’s modulus (E) and Poisson’s ratio (ν) for the EPAX hard and tough clear resin are used as input. Loads and constraints are shown in Figure 1. The loads associated with these conditions are 30 N distributed over the surface and 2 MPa of pressure distributed on the upper guide for the steel wire required for valve adjustment. In each analysis, the value of maximum vertical displacement (always recorded at the contact point between the cam at maximum opening and the end stop on the handlebar) is obtained, as shown in Figure 3.

The EPAX photopolymer resin was modeled as a homogeneous, isotropic, linear elastic material. The elastic properties were taken from the manufacturer’s technical datasheet:

- Young’s modulus: E=2.2 GPa;
- Poisson’s ratio: ν=0.38 (typical for photopolymer resins).

Given the small deformation regime investigated in this study, non-linear material effects (plasticity or viscoelasticity) were neglected.

The throttle cam was constrained at the central mounting region to reproduce the handlebar fixation condition. All translational degrees of freedom were restricted at the inner cylindrical surface, preventing rigid body motion.

A vertical concentrated load was applied at the external profile of the cam to simulate the cable-induced force at the end-of-travel configuration. The load magnitude corresponded to the operational range investigated in parametric study.

The loading condition was quasi-static, and displacement-controlled simulations were performed to evaluate the global structural response. The geometry was discretized using three-dimensional tetrahedral solid elements. Second-order quadratic elements were adopted to improve stress and displacement accuracy in curved regions. The maximum displacement and global stiffness were monitored as convergence indicators. Variations between the medium and fine meshes were below 2%, and therefore the medium mesh was selected as a compromise between accuracy and computational cost. However, this is not the focus of the dissertation. Users can replicate the experiment using the customized meshing strategy if it is maintained during the analysis of the various lattice configurations. The correct application of the RSM is the most important purpose, not the type of constraints and geometry or the refinement of the mesh used (however, this should be thin enough to represent the core, no more than 20% of the thickness of the core rods).

**Figure 4:**
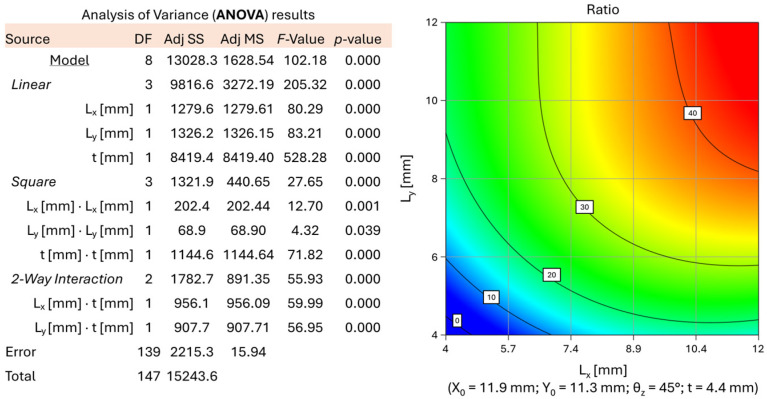
Results of the %_smc_ ANOVA (2nd order) and examples of sub-optimal sizing (definition of L_x_ and L_y_) as the other input parameters vary.

The specific value of stiffness analyzed in this study is here defined as the ratio r between the applied load (N) and the maximum displacement evaluated along the vertical direction. This is the objective function to maximize. The methodology proved effective, and optimal and sub-optimal design points were identified within the analysis domain. At these points, the structural stiffness-to-weight ratio is maximized. In the specific RSM case study, third- and second-order polynomial solutions are sufficient to achieve high compatibility between predicted data and the actual results obtained from the analyses. For RSM analysis, an a value of 0.05 is used to eliminate all the uninfluencing variables. Usually, a significance level (denoted as a) of 0.05 works well. A significance level of 0.05 indicates a 5% risk of concluding that an association exists when there is no actual association. Models from the sixth polynomial order down were considered, and the one of order 3 resulted as the most accurate and complete for the calculation of displacements thanks to the adjusted R^2^ coefficient of 0.9059 and the forecast R^2^ of 0.8012. Despite several attempts, it was not possible to improve these values. On the other hand, a 2nd order polynomial model was more accurate for the estimation of the percentage of unprinted mass in the core, with adjusted coefficients R^2^ equal to 0.9915 and predicted R^2^ equal to 0.9900. Some samples with excessive residues or Cook’s distance (references in literature) were reviewed or eliminated from the simulation. In some cases, values of θ close to 90° or too-low thickness values contributed to the average distortion of the model. The most influencing parameters were L_x_, L_y_ and t, i.e., the size of the cells and the trabecular thickness. This is confirmed by the low value of the *p*-value associated with them, as demonstrated in Figure 4. Maximum lightening is achieved with a completely empty core, and maximum throttle cam stiffness (as little displacement as possible) is obviously achieved with a fully filled core. Thanks to the mapping carried out on the analysis domain, however, it is possible to find the presence of maximum ratios (stiffness/weight) in points of optimal or sub-optimal design that can be evaluated during the design phase. As can be seen in Figure 4, it is possible to analyze the effect that the combined variation of one or more parameters has on the final ratio r of the structure.

**Figure 5:**
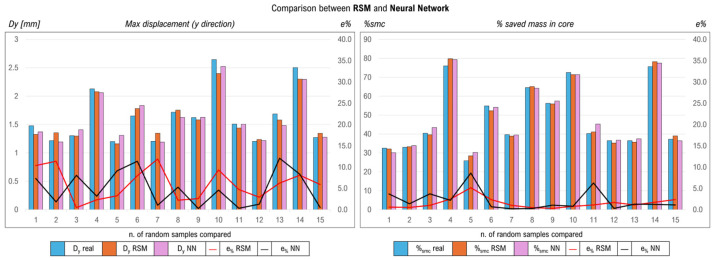
Comparison between RSM and neural network results.

For the NN training on the database, consisting of 250 samples, the choice of 10 neurons per layer followed an iteration process in which multiple configurations were trained on the same database. This configuration turned out to be the best. Alternative setups of the network (more layers, more neurons) can certainly improve its predictive ability and can be found by trial and error or by exploiting numerous iterations of the programmed network. Here, the proposed combination was the best among some setups analyzed (even with multiple neurons). The network was built using Matlab R2022b, leveraging Deep Learning packages. A total of 450 epochs with batch size of 45 were used, setting the learning rate at 0.01. It is very difficult to optimally configure a neural network, but post-training comparisons with the values in the database suggest good reliability and predictive capacity. This is also shown in Figure 5. The configuration of an optimal network is not the focus of the dissertation, as it serves to provide a concrete comparison to the RSM model. After 65 s, the network was trained. All analyses were performed on a device with a 2.8 GHz CPU, four cores and eight logical processors, 16 Gb of RAM and integrated NVDIA GeForce GTX 1050 GPU. A comparison between the RSM and NN results on several sets of samples allowed us to confirm the validity of both methodologies despite the high complexity of the model. Both models can accurately predict the displacement and lightening values, allowing good estimates of the final ratio. With more in-depth training, it is possible that the neural network improves its accuracy by surpassing the RSM, confirming some related studies already known in the literature [32,41]. A comparison of 15 random samples is shown in Figure 5. The vertical axes in the figure show the displacement and mass values saved on the left and the percentage of error calculated for each randomly selected sample on the right. This percentage of error is evaluated between the real value and the value predicted by the RSM and NN. The columns show real and expected values, while the lines show the percentage error value for each sample. It is possible that the neural network requires more training to improve the convergence of results. However, the results obtained are aligned, and the error rates are low.

### 3.1. Simple Physical Validation with Lab Experiments (Worst Load Application)

To address the main limitation associated with purely numerical and data-driven approaches, an experimental validation campaign was conducted on selected throttle cam configurations. The purpose of this experimental activity is not to provide a full mechanical characterization of the lattice material but rather to validate the stiffness metric adopted throughout the optimization process and to assess the predictive capability of the FEM, RSM, and ANN models under representative loading conditions.

A limited number of throttle cam specimens were manufactured using the same resin additive manufacturing process described in Section 3. Three representative lattice configurations were selected from the design space:

- One optimal configuration identified by the RSM analysis;
- One sub-optimal configuration located near a local maximum of the stiffness-to-weight ratio;
- One non-optimal configuration selected at the boundary of the analysis domain.

For each configuration, three identical specimens were printed, resulting in a total of nine tested components. All specimens were manufactured using the same EPAX resin, printing orientation, slicing parameters, and post-processing procedure (washing, support removal, and UV curing) to minimize process-induced variability. This choice allows isolating the effect of lattice geometry on the measured mechanical response.

**Figure 6:**
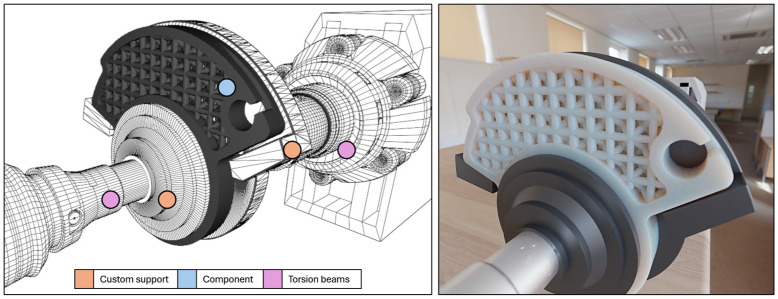
Configuration (CAD) of the cam in the torque transducer used to design custom supports and magnification on the thickness useful for transmitting force to the contact area.

Quasi-static mechanical tests can be performed using a generic torsion testing machine. The main problem concerns the need to design and manufacture two fixing flanges useful for simulating the cam limit switch, as in Figure 6.

The machine is equipped with a calibrated load cell. Then, a custom fixture was designed to reproduce the boundary conditions adopted in the numerical simulations. The throttle cam was rigidly constrained at its mounting region, preventing rigid body motions and rotations, while leaving the remaining portion of the component free to deform.

The load was applied vertically at the contact region corresponding to the end-stop condition of maximum throttle opening, consistent with the FEM setup described in Section 2.1. A flat loading indenter was used to apply force, ensuring repeatable contact conditions across specimens.

Each specimen was subjected to a displacement-controlled loading protocol at a constant crosshead speed of 1 mm/min, corresponding to quasi-static conditions. After an initial preload of 5 N to ensure full contact between the indenter and the component, the load was increased up to 30 N, matching the force level adopted in the numerical analyses.

Three consecutive loading–unloading cycles were applied to each specimen to account for possible viscoelastic effects of the resin material. Data from the third cycle were used for the evaluation of the structural response, as it exhibited stabilized force–displacement behavior.

During the tests, the applied force and the corresponding vertical displacement at the loading point were continuously recorded. The experimental stiffness $k_{exp}$ was defined consistently with the numerical model as (9):

(9) $$k_{exp}=\frac{F}{\delta }$$

where F is the applied load and δ is the measured vertical displacement. It should be noted that the values of force and displacement correspond to torque/angle ratios measured by the torque transducer and related to the average radius of the cam at the end of the switch. To reduce the influence of local non-linearities, stiffness was evaluated as the slope of the force–displacement curve obtained through linear regression in the 10–30 N load interval.

The experimentally measured displacements and stiffness values were compared with the corresponding predictions obtained from FEM simulations, RSM regression models, and ANN estimations. The comparison focuses on relative errors and trends rather than absolute values, as the primary objective of the study is the validation of the data-driven optimization framework.

This experimental validation provides a physical confirmation of the stiffness trends predicted by the numerical and machine learning approaches, supporting the reliability of the proposed methodology for the optimization of lattice-filled components under realistic loading conditions, as summarized in Table 1.

The load was applied using a flat indenter instead of reproducing the actual cable–cam contact geometry. This choice was intentionally adopted to ensure repeatability of the experimental measurements and direct consistency with the numerical model. Since the objective of the experimental campaign is the validation of global stiffness trends rather than a detailed investigation of local contact mechanics, the simplified contact condition is considered fully representative for the purposes of the study.

### 3.2. 3D Printing Phase—First Physical Prototyping

Once the optimal design was identified, a first resin prototype was manufactured and mounted on the motorbike handlebars for testing. The slicing software used for preparing the model for printing was Lychee, version 1.7. The cam was printed in a vertical orientation to improve the printing resolution, reducing warping issues beyond the symmetry plane. Manually designed supports were also used. First, they support undercut surfaces within some layers. Then, they prevent deformation due to material shrinkage, ensuring proper separation of the part from the FEP layer by applying adequate force. The pattern was uniformly scaled by 101% directly within the slicer to counteract shrinkage during the printing and curing phases. This adjustment was designed to compensate for potential dimensional variations in the printed and cured part. Printing settings, according to the manufacturer specification, are set as shown in the table below (Table 2).

**Figure 7:**
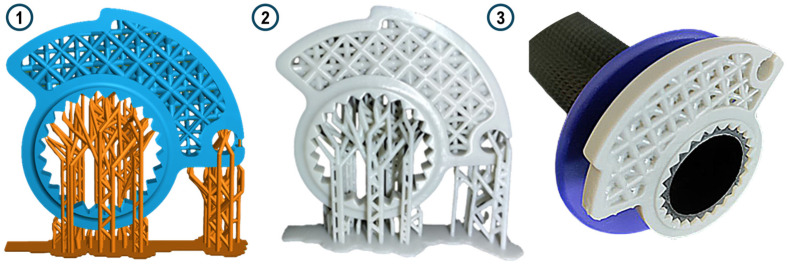
Prototyping steps: design of the throttle cam and supports, 3D printing with resin and first assembly test. (**1**) Display of pre-printing model; (**2**) printed part before removing supports; (**3**) manufactured component is mounted on the handlebars of the motorcycle.

**Figure 8:**
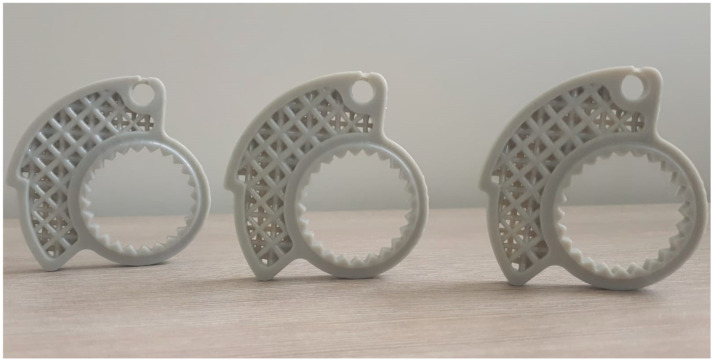
Three manufactured components.

The high exposure value of the base layers (24 s) ensures the best possible adhesion to the aluminum printing platform. Normal layers, on the other hand, have a shorter exposure time (2.5 s), thus ensuring maximum detail resolution and avoiding overexposure that would lead to deformation and failure to meet prescribed dimensional tolerances. Slicing of the component was done in “.ctb (v4)” format, which allows vertical movement of the head at two different speeds. A low speed is therefore maintained in Section 1, the one closest to the resin container, both in the ascent and approach phases. In contrast, the speed in Section 2 is much higher. This avoids breakage or detachment of the part from the build plate in the phase of detachment from the FEP and then increases the speed when detachment is complete. After printing, the component was washed in IPA (isopropyl alcohol) through an ultrasonic cleaner. This allows the removal of any excess resin trapped in the component or inside the lattice geometry. The component underwent a 20 min ultrasonic cleaning cycle, the time suggested by the resin producer. Next, a manual support removal step was performed with the use of a little cutter. A careful visual inspection was performed to assess macro-defects such as failure of some parts of the structure, delamination or breakage occurring after the removal of the supports. The final step is the curing process to complete the polymerization of the part. Curing was performed using the XYZ printing 180 Multicure station. Following the manufacturer’s directions, a curing time of 15 min was set with UV lights ranging between 385 and 405 nm at 120 W. In Figure 7, the design of the printing supports, the first printed prototype, and an example of the mounting on the handlebar are shown for greater clarity. In Figure 8, three prototypes are photographed to show the geometry and final appearance to readers.

## 4. Conclusions

In conclusion, the authors confirm that both approaches were valid for the analysis of the parameterized core and for the identification of the optimal and sub-optimal design points. The second- and third-order RSM models and the neural network can all predict with good precision the design ratios between saved mass and measured displacement in the throttle cam limit switch area. As there are two different methodologies applied to the same dataset, we can confirm that they can be integrated for optimization studies, and one validates the other. The predicted results are good considering the complexity of the model and its parameterization. In particular, the neural network can be integrated as a component of a more complex network capable of predicting these outputs also considering different geometries, loads and constraints. The proposed approach is in both cases considered appropriate as a reference guide for the design of core lattices avoiding unruly applications, as still happens despite the numerous scientific works concerning this topic.

## Figures and Tables

**Table 1:** Comparison between experimentally measured displacements and corresponding predictions obtained from finite element simulations, Response Surface Methodology (RSM), and Artificial Neural Networks (ANNs) for representative throttle cam lattice configurations. Experimental values are reported as mean ± standard deviation over three specimens. In columns are listed: configuration, FEM displacement, experimental displacement, RSM displacement, and ANN displacement of different configurations demonstrating the good predictive capability of both RSM and ANN models at least before the first yield (pure elastic behavior).

Config.	δFEM (mm)	δEXP (mm)	δRSM (mm)	δANN (mm)	Error RSM (%)	Error ANN (%)
Optimal	0.42	0.45 ± 0.02	0.44	0.43	2.2	4.4
Sub-optimal	0.57	0.61 ± 0.03	0.59	0.58	3.3	4.9
Non-optimal	0.81	0.86 ± 0.04	0.84	0.82	2.3	4.7

**Table 2:** 3D printing settings, according to manufacturer specification.

Burn in Layers (4 Layers)			Normal Layers		
Exposure time	24	s	Exposure time	2.5	s
Lift distance (1)	4	mm	Lift distance (1)	4	mm
Lift distance (2)	3	mm	Lift distance (2)	3	mm
Retraction distance (2)	4	mm	Retraction distance (2)	4	mm
Retraction distance (1)	3	mm	Retraction distance (1)	3	mm
Lift speed (1)	50	mm/min	Lift speed (1)	50	mm/min
Lift speed (2)	150	mm/min	Lift speed (2)	150	mm/min
Retract speed (2)	150	mm/min	Retract speed (2)	150	mm/min
Retraction speed (1)	50	mm/min	Retraction speed (1)	50	mm/min

## Data Availability

The raw data supporting the conclusions of this article will be made available by the authors upon request.

## Abbreviations

The following abbreviations are used in this manuscript:

RSM	Response Surface Methodology
CAD	Cad-Aided Design
ANOVA	Analysis of Variance
CCD	Central Composite Design
LHS	Latin Hypercube Sampling
ANN	Artificial Neural Network
ML	Machine Learning
UV	Ultraviolet
FEP	Fluorinated Ethylene Propylene
IPA	Isopropyl Alcohol
